# Supplementary material for: FADE-CTP: A Framework for the Analysis and Design of Educational Computational Thinking Problems
Source: Technol Knowl Learn. 2025 Mar 10;30(2):1073–148. doi: 10.1007/s10758-025-09833-x (PMC12124242; doi:10.1007/s10758-025-09833-x)
Supplement: Supplementary file 1 — (PDF 33 KB) [file 10758_2025_9833_MOESM1_ESM.pdf]

Supplementary information

Explaining the connection between CTP characteristics and CT competencies in the FADE-CTP framework

Giorgia Adorni · Alberto Piatti · Engin Bumbacher · Lucio Negrini · Francesco Mondada · Dorit Assaf · Francesca Mangili · Luca Gambardella

This document provides a detailed analysis of our framework, FADE-CTP, illustrating the rationale behind mapping CTP characteristics to CT competencies.

We have organised this analysis according to the main levels of the activity dimension: problem setting, algorithm, and assessment. For each dimension, we first describe the link between skills and the required features, then the link between the skills and the characteristics that act as catalysts.

The CTP features we considered are the functionalities allowed to the problem solver by the tools, the property of the system, such as resettability and observability, and finally, the trait of the task, including the elements required to be found, the type of cardinality of the elements given and to be found, the presence of constraints and the type of representation of the algorithm.

G. Adorni · F. Mangili · L. Gambardella
Dalle Molle Institute for Artificial Intelligence (IDSIA), USI-SUPSI, Lugano, Switzerland

A. Piatti · L. Negrini
Department of Education and Learning (DFA), SUPSI, Locarno, Switzerland

E. Bumbacher
HEP-VD, Lausanne, Switzerland

F. Mondada
Mobile Robotic Systems Group (MOBOTS), EPFL, Lausanne, Switzerland

D. Assaf
School of Education, FHNW, Windisch, Switzerland

Table 1 Comprehensive overview of the relationship between different CTP characteristics and CT competencies. The table shows the relationship between the characteristics of CTPs (columns) and CT competencies (rows). The CTP features considered include the tools' functionalities, the system's properties, and the task trait.

		**Tool functionalities**								**System**				**Task**									
		Variables	*Operators*	*Sequences*	*Repetitions*	*Conditionals*	*Functions*	*Parallelism*	*Events*	*System resettable*	*System not resettable*	*System (partially) observable*	*System not observable*	*One-to-one cardinality*	*Many-to-one cardinality*	*Explicit elements*	*Implicit elements*	*Unconstrained elements*	*Constrained elements*	*Algorithm manifest*	*Algorithm latent*	*Algorithm written*	*Algorithm not written*
**Problem setting**	*Data collection*	✓							+	+	+	+	+	+	+		+		+	+	+	+	+
	Pattern recognition	+		+	✓*	+	✓*	+	+	+	+			+	+	+	+	+	+	+	+	+	+
	Decomposition	+	+	✓*	+	+	✓*	+		+	+		+	+	+	+	+	+	+	+	+	+	+
	Abstraction	✓		+	+	+	✓				+		+		+	+	+	+	+	+	+	+	+
	Data representation	✓		+	+	+	+				+		+		+		+		+	+	+	+	+
**Algorithm**	*Variables*	✓	+	+	+	+	+	+	+			+	+	+	+	+	+	+	+	+		+	+
	Operators	+	✓	+	+	+	+	+	+			+	+	+		+	+	+	+	+		+	+
	Sequences	+	+	✓	+		+					+	+			+	+	+	+	+		+	+
	Repetitions	+	+	+	✓		+					+			+	+	+	+	+	+		+	+
	Conditionals	+	+			✓			+			+	+	+	+	+	+	+	+	+		+	+
	Functions	+	+	+	+		✓					+			+	+	+	+	+	+		+	+
	Parallelism	+	+					✓				+			+	+		+		+		+	+
	Events	+	+			+			✓			+				+		+		+		+	+
**Assessment**	*Algorithm debugging*	+	+	+	+	+	+	+	+	✓	✗	+			✓				+		✗	✓	✗
	System state verification									✓	✗	+					+			✓	✗	✓	✗
	Constraints validation	+	+	+	+	+	+	+	+	✓	✗	+					✗	✓					
	Optimisation	+	+	+	+	+	+	+	+	✓	✗	+											
	Generalisation	✓		+	+	+	✓		+	✓	✗	+			+		+		+				

✓ indicates that the characteristics is required for the development of the competence.
✓* indicates that at least one of several characteristics in a group is required for the development of the competence.
✗ indicates that the characteristic prevent the development of the competence.
\+ indicates that the characteristic can support the development of the competence.
Blank cells indicate that the characteristic is irrelevant for the development of the competence.

1 Problem setting competencies

1.1 Characteristics required for competencies development

Starting from the problem setting skills, to activate the "data collection" competence, the only requirement is that the tools available allow the use and recognition of variables. Without variables, there would be nothing to collect data on. The "pattern recognition" competence requires the presence of repetitions or functions since they allow the identification of repeating patterns in the data. The "decomposition" competence requires the presence of functions or sequences that can be used to break down a complex problem into smaller more manageable components. The "abstraction" competence demands the presence of variables to represent key concepts and functions to encapsulate and reuse specific behaviour within a single, self-contained unit, simplifying the original task and allowing the problem solver to reason about the problem at a higher level of abstraction. Finally, the "data representation" competence requires only variables to represent data.

1.2 Characteristics supporting competencies development

Generally, the attributes of the problem not required directly to activate the skills can influence them in some way. In the case of the characteristics of the tools, for example, variables also play a role in "pattern recognition" and "decomposition", as they can be used to store patterns or parts of a complex problem. Then, operators can be useful for the "decomposition" of the problem into smaller parts. At the same time, sequences

can contribute to the processes of "pattern recognition" and "abstraction", helping the problem solver to identify patterns or regularities in the data, as well as the key concepts or essential elements of a problem, but also in "data representation" to organise and present data in a clear and meaningful way. Repetitions can influence the activation of the problem setting skills of "decomposition", "abstraction", and "data representation" because they can make the task more complex thus requiring the problem solver to use these practices. Similarly, conditionals can help to structure and simplify a problem, making it more manageable and easier to solve, enabling "pattern recognition", "decomposition", "abstraction" and "data representation". Functions can influence the activation of "data representation" by helping the problem solver organise and structure data. Parallelism can influence the activation of the problem-solving skill of "decomposition" as it allows for breaking the problem into independent subtasks that can be executed simultaneously. Finally, events can trigger "data collection" at a specific point in time. For the sake of the characteristic of the system, resettability allows the problem solver to start over and try different approaches to solving the problem, thus stimulating can problem setting skills such as "data collection", "pattern recognition", and "decomposition", as they can test different strategies and collect data on their effectiveness. On the other hand, if the system is not resettable, the problem solver may have to rely more on "abstraction" and "data representation" skills to find a solution, as they cannot try different approaches and must work with the information they have available. In general, a resettable system allows more freedom for the problem solver, giving a chance to explore different solutions. In contrast, a non-resettable system may require more creativity to find a solution. If the system is observable, the problem solver would likely use skills related to "data collection", as he can directly perceive and then gather information about the system's state and properties. Additionally, he may use skills related to "pattern recognition", such as identifying patterns or trends in the data collected. These skills can help the problem solver understand the system's current state and make informed decisions about how to solve the task. Conversely, suppose the system is not observable. In that case, the problem solver may need to rely on abstract and hypothetical reasoning to devise a solution, activating "pattern recognition", "decomposition", and "abstraction" to understand the problem and identify possible solutions. Also "data collection" may be necessary to gather information about the system and its behaviour, even if that information is not directly observable. Additionally, the "data representation" skill can be used to organise and interpret the information they have collected to make sense of the problem and develop a solution. When there is a many-to-one cardinality in the system, it means that there is a large amount of data that needs to be processed, and multiple inputs or sources of information can be used to achieve a single goal or outcome. In this scenario, the "data collection" skill will likely be activated because the problem solver needs to gather a large amount of information to understand the problem and find a solution. Since there are multiple instances of a certain element or pattern, recognising the commonalities and differences among them would be essential to understand the overall system, leading to the use of more complex data collection and analysis strategies, thereby activating the "pattern recognition" competence. The "decomposition" and the "abstraction" skills will also likely be activated as the problem solver needs to break down the problem into smaller manageable parts and find the underlying principles and concepts in the problem to understand the overall system and find a solution. Finally, as there are multiple instances of a specific element, it would be essential to communicate them clearly and concisely, thus activating the "data representation" skill. By contrast, if there is a one-to-one cardinality in the system, the competencies of problem setting that are likely to be activated include "data collection", "pattern recognition", and "decomposition". The skills "abstraction" and "data representation" are less likely to be activated since the direct correspondence between the system elements means there is less need to abstract or represent the information. It can be assumed that with implicit elements, the "data collection", "pattern recognition", "decomposition" and "abstraction" competencies may be activated as the problem solver needs to infer information from the context or the environment, understand the underlying concepts or patterns in the task, decompose the problem into smaller sub-problems, and create abstract representations of the system. The same reasoning can be applied to constrained elements. Moreover, it is possible that the competence "data representation" may be activated as implicit or constrained elements may require the problem solver to think about how to represent the data in a way that accurately reflects the underlying information or constraints. Likewise, with explicit elements, the "pattern recognition", "decomposition", and "abstraction" competencies may also be activated, as the problem solver needs to understand and make sense of the given information, and the presence of unconstrained elements to be found may allow for more flexibility and creativity in problem-solving, potentially activating these skills, as problem solvers may need to find novel ways to organise or make connec-

tions among the elements. In this scenario, the "data collection" and "data representation" competencies may be more straightforward and not as crucial, especially when the elements are explicit and thus the information is already provided in a structured format. Regarding the representation of the algorithm, overall all problem setting competencies may be activated. Nevertheless, a manifest algorithm makes the problem solver's task easier by providing a clear set of instructions and reducing the need for "pattern recognition" and "decomposition". However, a not manifest algorithm can promote more "pattern recognition", "decomposition", and "abstraction" as the problem solver needs to infer the algorithm from the problem statement and available information and cannot represent it.

2 Algorithmic competencies

2.1 Characteristics required for competencies development

For the algorithm dimension, each competence to be activated requires that the corresponding characteristic of the tool is enabled. For example, to activate the "variable" skill, the tools used by the problem solver should include variables. Moreover, in a formal artefactual environment, the task requires that the algorithm is not given but has to be found. Otherwise, it is possible only to assess the problem solver ability to recognise these skills and apply them, but not create an algorithm from scratch.

2.2 Characteristics supporting competencies development

Again, some characteristics can also influence the activation of algorithmic competencies. Regarding the characteristics of the tools, for example, the presence of variables may influence the activation of all the other algorithmic skills, since they provide a fundamental building block for creating algorithms and can be used in conjunction with other algorithmic structures. Similarly, operators influence the activation of all algorithmic skills. The presence of sequences may influence the activation of "variables", "operators", "repetitions" and "functions"; repetitions may influence the activation of "variables", "operators", "sequences" and "functions"; the presence of conditionals may influence the activation of "variables", "operators" and "events"; functions may influence the activation of "variables", "operators", "sequences" and "repetitions"; the presence of parallelism may influence the activation of "variables" and "operators"; while events may influence the act "variables", "operators" and "conditionals". The resettability or non-resettability of a system is not relevant for activating or not algorithmic competencies. The system's observability, or the ability to observe the agent's actions and the system's state, allows tracking of how the algorithm is executing and makes it easier for the problem solver to identify these procedures used by the agent. Instead, a non-observable system may activate the skills of "variables", "operators", "sequences", and "conditionals", since the problem solver may need to rely more heavily on their ability to reason about the system and make inferences based on limited information. Regarding the ratio of elements given and to be found, from one side, a one-to-one cardinality may influence the activation of the algorithmic skill "variables" that can be used to define the direct correspondence between the elements in the system and their representations, but also of "operators" and "conditionals" proper to manipulate them and necessary to ensure the correct mapping. On the other side, a many-to-one cardinality can make it more challenging to understand the relationship between the given elements and those to be found, impacting the ability to understand the algorithm and its parts and enforcing the use of certain types of structures. For example, the problem solver can keep track and map multiple instances to a single object using "variables". If the task at hand involves processing multiple pieces of data and producing a single result, a "repetition" can be used to iterate over the inputs. Similarly, "conditionals" can be used if the task requires selecting one output out of multiple possibilities based on certain conditions. In contrast "functions" can be used to modularise the code and make it more organised and maintainable. Finally, "parallelism" can be used to speed up the processing of multiple inputs by running multiple iterations simultaneously. Further, explicit elements provide clear and specific information about the task that must be solved, allowing the problem solver to use all the algorithmic structures to manipulate and work with that information to achieve the desired outcome. Besides, the presence of implicit elements in the task makes it more difficult for the problem solver to understand and determine the necessary steps to solve the task, thus some algorithmic structures may need to be used to compensate for this shortcoming. For example, "variables" would be necessary to store and track the values of implicit elements, "operators", "sequences", "repetitions", "conditionals", and "functions" would help make decisions and perform actions based on the values of these variables. These algorithmic structures would allow the problem solver to explain the implicit elements effectively and develop more sophisticated and efficient

solutions. Similarly, the space for possible solutions is limited when constrained elements are involved in the task and it may be necessary to use some algorithmic structures to ensure those constraints are met. For example, while solving a puzzle, the final state and algorithm have to be found, and they have constraints: the problem solver has to fit several pieces together to form a complete image, pieces must fit together to form a specific figure, and certain pieces can only be placed in certain orientations. To solve this task, the problem solver might use a combination of algorithmic structures such as "variables" to keep track of the current state of the puzzle and the position of the pieces, "operators" to manipulate the pieces and move them around, "sequences" to try different combinations of pieces, "repetitions" to keep trying different combinations until the puzzle is complete, and "conditionals" to check if the current combination of pieces meets the constraints. Additionally, "functions" could also be used to group sets of repeated actions. Finally, how the algorithm is represented can affect the activation of various algorithmic structures depending on the type of representation used. Considering different types of tools, each can be more suited to activating one skill rather than another. If the algorithm is represented in a mathematical notation, the use of "operators" may be more prominent. On the one hand, if the algorithm is represented in a visual block-based programming language, the use of "sequences", "repetitions" and "conditionals" may be more intuitive and easier to activate. On the other hand, if the algorithm is represented in a text-based programming language, the use of "variables" and "functions" may be more natural to activate. Finally, robotic programming languages are usually designed for detecting and responding to "events", such as sensor readings or other inputs. They often have built-in functionalities for concurrent execution of multiple instructions, allowing "parallelism". Overall, the choice of representation can affect the ease and familiarity of activating different algorithmic structures and may also shape the problem solver's understanding and ability to apply them effectively.

3 Assessment competencies

3.1 Characteristics required for competencies development

Finally, in the assessment category, all skills have in common the need for the system to be resettable for the skill to be activated. For example, in "algorithm debugging", if the instruction cannot be reversed, it is impossible to revise and test the previous code versions. Thus, resettability is necessary to debug the algorithm in a controlled and repeatable environment. The same applies to correcting errors in the state and constraints and improving the solution's performance or generalising it. In the specific case of "algorithm debugging", this skill can be activated in all the artefactual environments if the algorithm has to be found and if it is manifest because it allows the user to understand and check the logic and the flow of the algorithm. This is essential to identify and fix any bugs or errors in the algorithm. While it becomes increasingly important to have a written algorithm as the difficulty level of the artifactual environment rises, it may still be possible to solve the problem without one. However, the absence of a written algorithm may make it more challenging to analyse or modify the solution in a formal setting, as the artefactual environment is more abstract and requires a more in-depth understanding. For this reason, we considered the skill required in this context. The "system state verification" competence can be activated in all three artefactual environments if at least one between the initial and final states must be found. In embodied environments, direct physical interactions with the system provide a way to observe its state without needing a manifest algorithm. However, in symbolic and formal environments, a manifest representation of the algorithm, written in the case of formal environments, is crucial to fully understand its logical flow, verify the system state, and perform formal reasoning about its correctness. This may involve analysing the symbolic representation to understand how it impacts the system state. To activate the "constraints validation" competence, it is blatant that the other necessary characteristic is having constraints on the states to be found. To enable "optimisation", additional features are not required, while for "generalisation", variables and functions are necessary to reuse and apply the task solution to different problems.

3.2 Characteristics supporting competencies development

Each tool functionality available to the problem solver can be a potential cause of error in the algorithm. For example, if the problem solver is unfamiliar with one of them or does not understand how to use it correctly, he may not use it at all or misuse it. This can lead to errors in the algorithm and potentially result in the problem not being solved correctly. This is why functionalities of the tools if available can activate "algorithm debugging". Also for "constraints validation", all the characteristics of the tools are influential. Above all, variables, operators, conditional and functions may

allow the problem solver to perform various calculations and comparisons to check if the values assigned to the variables meet the specified constraints. Further, it could be that the constraint imposed is precisely on the algorithm and prohibits using some of these structures. The functionality of the tools available to the problem solver can greatly impact the "optimisation" of the algorithm in several ways. Parallelism allows for multiple tasks or processes to be executed simultaneously, which can greatly reduce the overall time required to complete a task. Sequences and other structures, such as loops, can also help to improve efficiency by allowing for the automation of repetitive tasks and the ability to perform actions in a specific order. Additionally, using functions and subroutines can improve the readability and maintainability of the algorithm, making it easier to identify and fix any errors that may occur. However, having access to a wide range of functionalities can make it challenging for the problem solver to choose the appropriate one for a specific task, leading to a revision of the solution to increase efficiency and performance. The competence "generalisation" can also be influenced by other characteristics of the tools. The presence of sequences and repetitions in the toolset enables the problem solver to apply the same algorithm to different parts of a problem or task. Similarly, the inclusion of conditionals allows for the application of different algorithms depending on the specific conditions of the task. Furthermore, the presence of events in the toolset allows for creating algorithms that can respond to different triggers within the problem, leading to a greater generalisation of the solution and the ability to adapt to changes within the problem. In terms of observability, an observable system allows the problem solver to have a clear understanding of the system's state and the output of the algorithm, which can aid in identifying and addressing errors and inefficiencies and performance issues, as well as recognising patterns or regularities that can be generalised to new or different situations. However, it is essential to note that while observability can aid in all assessment skills, it is not strictly necessary for their activation. For example, one could still perform "algorithm debugging" and "system state verification" on a non-observable system, though it may be more difficult. Similarly, "generalisation" can still occur without perfect observability, but it may be harder to identify patterns and regularities without direct access to the system state. If the system has a many-to-one cardinality, the competence of "generalisation" may be activated as it would be necessary to apply the same algorithm to different inputs or outputs. If the system contains implicit elements, the competencies "algorithm debugging" and "system state verification" may be activated as the problem solver may need to identify and troubleshoot any issues with the algorithm that are not immediately apparent or infer the current state of the system based on the implicit information provided. Also "generalisation" may be activated as the problem solver may need to apply the algorithm to different situations based on the implicit information provided. Finally, suppose in the system there are elements to be found with constraints. In that case, the "generalisation" skill may be activated because it requires the problem solver to adapt the task to the specific constraints and can be intended as solving a new problem using the knowledge acquired in a previous situation and adapting it to a new one.

Fundings

This research was funded by the Swiss National Science Foundation (SNSF) under the National Research Program 77 (NRP-77) Digital Transformation (project number 407740_187246).

Competing Interests

The authors declare that they have no conflict of interest.

Author Contributions

Giorgia Adorni: Conceptualization, Methodology, Validation, Formal analysis, Investigation, Resources, Data curation, Writing - original draft & review & editing, Visualization, Supervision.
Alberto Piatti: Conceptualization, Methodology, Validation, Formal analysis, Writing - original draft & review & editing, Supervision, Project administration, Funding acquisition.
Engin Bumbacher: Conceptualization, Methodology, Validation, Formal analysis, Writing - original draft & review & editing.
Lucio Negrini: Validation, Formal analysis, Investigation, Writing - original draft & review & editing.
Francesco Mondada: Validation, Visualization, Writing - review & editing, Supervision, Project administration, Funding acquisition.
Dorit Assaf: Writing - review & editing, Funding acquisition.
Francesca Mangili: Writing - review & editing.
Luca Maria Gambardella: Funding acquisition.
